# Interannual variation in spring weather conditions as a driver of spring wildflower coverage: a 15-year perspective from an old-growth temperate forest

**DOI:** 10.1093/aobpla/plad078

**Published:** 2023-11-14

**Authors:** Lydia V Jahn, Sarah R Carrino-Kyker, David J Burke

**Affiliations:** The Holden Arboretum, 9500 Sperry Road, Kirtland OH, USA; The Holden Arboretum, 9500 Sperry Road, Kirtland OH, USA; The Holden Arboretum, 9500 Sperry Road, Kirtland OH, USA

**Keywords:** *Allium tricoccum*, *Cardamine concatenata*, *Claytonia virginica*, climate change, *Dicentra canadensis*, *Erythronium americanum*, precipitation, temperature

## Abstract

Spring ephemerals are wildflowers found in temperate deciduous forests that typically display aboveground shoots for a period of 2 months or less. Early spring, before the canopy leaves out, marks the beginning of the aboveground growth period where ephemerals acquire nutrients and resources via aboveground tissues. Several studies have shown that spring ephemeral reproduction is affected by spring temperature, but few have looked at how weather conditions of the current and previous seasons, including precipitation and temperature, influence aboveground growth. Here, we examine the response of a spring ephemeral community in a temperate hardwood forest to weather conditions during their current and previous growing seasons. For 15 years we estimated percent cover of each species within our community. We highlighted five dominant spring ephemerals within this community: wild leek (*Allium tricoccum*), cutleaf toothwort (*Cardamine concatenata*), spring beauty (*Claytonia virginica*), squirrel corn (*Dicentra canadensis*) and trout lily (*Erythronium americanum*). We compared changes in cover on both a community and species level from 1 year to the next with average precipitation and temperature of the year of measurement as well as the year prior. We found precipitation and temperature influence a change in cover at the community and species level, but the strength of that influence varies by species. There were few significant correlations between plant cover in the current year and temperature and precipitation in the 30 days preceding measurement. However, we found significant correlations between plant cover and precipitation and temperature during the previous spring; precipitation and cover change were positively correlated, whereas temperature and cover change were negatively correlated. Overall, cooler, wetter springs lead to an increase in aboveground cover the next year. Learning how individual species within a forest plant community respond to weather conditions is a crucial part of understanding how plant communities will respond to climate change.

## Teaser

We examined the response of a spring ephemeral community in a temperate hardwood forest to weather conditions during their current and previous growing seasons. For 15 years we estimated the percent cover of each species within our community. We compared changes in cover on both a community and species level from 1 year to the next with average precipitation and temperature of the year of measurement as well as the year prior. There were few significant correlations between plant cover in the current year and temperature and precipitation in the 30 days preceding measurement. However, we found significant correlations between plant cover and precipitation and temperature during the previous spring; precipitation and cover change were positively correlated, whereas temperature and cover change were negatively correlated. Overall, cooler, wetter springs lead to an increase in aboveground ephemeral cover the next year.

## Introduction

Spring ephemerals are wildflowers found in temperate deciduous forests that typically display aboveground shoots for a period of 2 months or less (Lapointe and Lerat 2006). This growth usually occurs after the snow has melted but before the tree canopy has developed, as the plants take advantage of the high light available during the spring season (Lapointe and Lerat 2006). Spring ephemerals have slightly varied growth cycles among individual species, but overall, they share the same basic growth patterns. Early spring, before canopy leaf out, marks the beginning of the aboveground growth period (i.e. the epigeous growth period) where ephemerals acquire nutrients and resources via leaves and aboveground tissues (Lapointe 2001). These resources will be used for growth the following year and are stored in their perennial organ (Lapointe 2001). At the end of spring, when the tree canopy is starting to close, both above and belowground tissues senesce (Lapointe 2001). The dormancy period continues until August when belowground tissues such as roots and buds begin to grow. This hypogeous growth is slow and continues until the following early spring when aboveground tissues appear again (Lapointe 2001).

Spring ephemerals primarily acquire carbon, CO_2_ and nutrients during the epigeous growth period in early spring. *Trillium erectum*, for example, collects and stores carbon in its rhizome for use the following spring (Routhier and Lapointe 2002). *Erythronium americanum* absorbs the majority of its carbon and nutrients during spring when aboveground tissue is present (Lapointe and Lerat 2006). *Dicentra canadensis* possesses a perennial organ with three modules that develop at separate times, 1 year after the other, during the aboveground growth cycle of the plant (Lapointe 2001). The first module produces flowers and leaves for the year prior, the second module produces flowers and leaves for the current year, and the third module will flower and leaf out the following year (Lapointe 2001). Once aboveground growth senesces, the first module dies. A new module grows in its place, using the carbon and nutrients gained from that spring, which will produce leaves and flowers in 2 years (Lapointe 2001). Consequently, the weather conditions spring ephemerals experience will have a direct impact on their future growth, be it one or several years in the future.

Two important climate variables for spring ephemerals are temperature and precipitation. *E. americanum* benefits in terms of biomass from lower temperatures during the epigeous growth period (Lapointe and Lerat 2006). *E. americanum* also develops most of its roots during colder months in the late fall, (Brundrett and Kendrick 1989), as does *Allium tricoccum* (Nault and Gagnon 1988). *Trillium ovatum* flowering is delayed by high levels of precipitation during the late winter and early spring months (Matthews and Mazer 2016). The contrasts between these variables and their effects on spring ephemerals raise important questions about the relationship between temperature, precipitation and spring ephemeral epigeous growth.

Past studies have examined the effects of temperature on the emergence of spring ephemeral aboveground tissue (Routhier and Lapointe 2002), flowering phenology (Matthews and Mazer 2016; Petrauski *et al*. 2019) and plant biomass (Lapointe and Lerat 2006; Badri *et al.* 2007). All concluded that warmer spring temperatures cause earlier development, including flowering, but that biomass benefits from cooler temperatures. The relationship between precipitation and spring ephemeral growth seems to be incomplete in the literature. Matthews and Mazer (2016) reported delays in flowering with higher precipitation levels in Western North America, but most other spring ephemeral precipitation research has focused on desert climates (Chen *et al.* 2019; Jia *et al.* 2019; Rominger *et al.* 2019; Mu *et al.* 2021). Despite the depth of research into the effects of temperature on spring ephemeral reproductive responses, no studies to our knowledge have examined how weather conditions of past seasons, including both temperature and precipitation, influence a change in aboveground cover for the following year. Many of these studies stop measurements at the end of a single growing season to study how biomass or leaf area changed over the span of 1 year. These methods, while informative, may not reveal the full extent of the impacts of interannual weather conditions on spring ephemeral growth. Long-term observational studies are helpful in assessing these impacts and are lacking in the literature. To our knowledge, studies that took place over more than just 1 year, such as Schemske *et al.* (1978), investigated only the weather’s relationship to epigeous growth within the same year. Lapointe (2001) briefly mentions unpublished data that would be an exception to this. Lapointe surveyed *E. americanum* over 5 years and found increases and decreases in total leaf area but does not mention how the weather conditions impacted ephemeral growth.

North American deciduous canopy trees are sensitive to warm spring temperatures and leaf out earlier under these conditions (Routhier and Lapointe 2002). Understory plant species that overwinter underground, such as spring ephemerals, might not be able to respond to these changes in spring weather (Heberling *et al*. 2019; Lee *et al.* 2022). This may result in a phenological mismatch between spring ephemerals and the tree canopy because decreased light availability to understory plant species would limit the carbon acquisition window for spring wildflowers (Lee *et al.* 2022). In order to further understand this relationship, we studied the change in percent coverage of a spring ephemeral community and five dominant species within a temperate hardwood forest in northeastern Ohio, USA. Examining cover each year, especially over a long period of time, will allow for an observation of general patterns in the spring ephemerals’ response to climate.

## Materials and Methods

### Site description and herbaceous cover sampling

**Figure 1.**
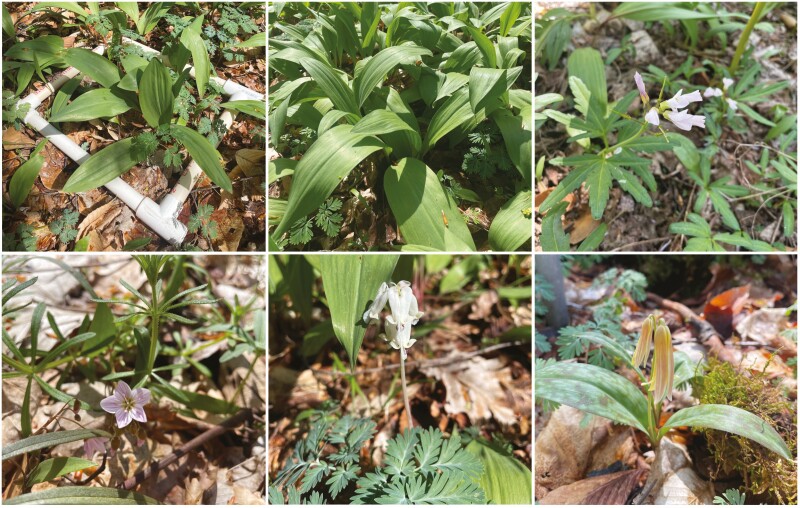
Spring ephemerals were included in this study and found at Stebbins Gulch. Upper left: Our 0.25 m × 0.25 m sampling plots; Upper middle: *A. tricoccum*; Upper right: *Cardamine concatenata*; Lower left: *Claytonia virginica*; Lower middle: *D. canadensis*; Lower right: *E. americanum*. Photos taken 19 April 2023.

**Figure 2.**
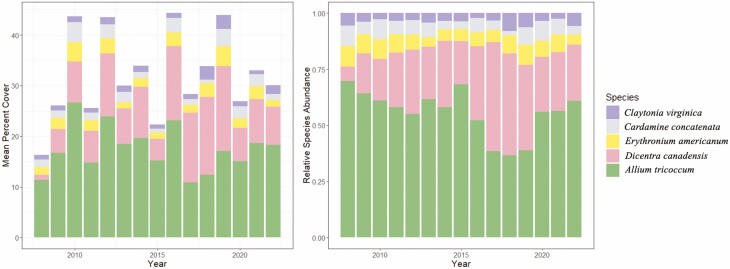
Mean percent cover (left) and relative species abundance (right) over time. In 2022, approximately 30 % of the forest floor was covered with spring ephemerals and nearly 60 % of that cover was comprised of *A. tricoccum.*

The study site, known as Stebbins Gulch, is a mature 360-ha hardwood temperate forest located at the Holden Arboretum in northeastern Ohio, USA (41° 36ʹ N and 81° 16ʹ W). The study site is located within an approximately 80-ha area of old growth (>200 years old). The herbaceous understory is largely comprised of spring ephemerals, with *A. tricoccum* (wild leek) and *D. canadensis* (squirrel corn) being the most abundant (Figs 1 and 2). The tree canopy is dominated by American beech (*Fagus grandifolia* ~ 75 %) and sugar maple (*Acer saccharum* ~ 15 %). Soils at the site consist of moderately drained, silt loam soils with acidic pH ranging from 3.5 to 5.6, with a mean pH of 4.0 ± 0.1. Total precipitation is approximately 116 cm per year, with approximately 287 cm of snowfall on average. The old-growth forest stands of beech, maple and mature hemlock led the US Department of the Interior to designate Stebbins Gulch as a National Natural Landmark in 1967 (https://www.nps.gov/subjects/nnlandmarks/site.htm?Site=HOLD-OH).

We established six long-term sampling plots in 2006 in an approximately 2.0-ha portion of mature beech-maple forest. The six sampling plots measure 4 × 4 m in size and are 50- to 100-m apart, with the centre of each plot marked by a 2-m steel pole driven into the ground (Supporting Information—Fig. S1). Four herbaceous plant sampling quadrats were established within each plot at four cardinal directions from the centre pole such that sampling plots are 1.0 m from the plot centre. Each of the four sampling quadrats in each plot measured 0.25 × 0.25 m (Fig. 1). Spring ephemerals were censused each spring beginning in 2008, between April 13 and May 2. Prior to every annual census, spring ephemeral leaf expansion was monitored visually by the same two individuals beginning in early March. Once leaf expansion was believed to be at its maximum, a formal cover survey was conducted within 2 days. The percent cover of each species present in the quadrat was estimated visually. All surveys were conducted by the same group of three people who conferred with one another to make sure their estimations were accurate and consistent. The study area and established monitoring plots have been the site of previous work exploring the ecology and distribution of mycorrhizal fungi (e.g. Burke 2015; Hewins *et al.* 2015; Burke *et al.* 2019).

### Weather data

Temperature and precipitation data were obtained from the National Oceanic and Atmospheric Administration (NOAA) Online Weather Data—Painesville Station. In each year from 2008 to 2022, we examined average temperature and average precipitation from the daily almanac data. Some of the data were missing from the daily almanacs due to broken sensors or power outages (NOAA 2023). If a precipitation measurement was missing, it was recorded as 0 inches of precipitation that day. If a temperature reading was missing, the average temperature for that day was generated by averaging the temperatures of the day prior and the next day. Once the data set was complete, we averaged the precipitation and temperature data across the 62-day period of March 15—May 15 to obtain the average spring weather conditions for each year. We also averaged the precipitation and temperature data for 30 days prior to the survey date in each year, with day 30 being the date the survey was conducted.

### Cover and change in cover calculations

The percent cover from each sub-plot was averaged at each of the six plots for the five dominant plant species within each year. This percent cover value was then analysed in response to weather conditions in the year of measurement. For example, the estimated percent cover in 2022 was analysed in response to the average temperature and precipitation (see above) from 2022 (i.e. the year of measurement). Further, the change in percent cover was calculated for each year interval, from 2008 to 2022, by subtracting the percent cover in the year of measurement from the percent cover in the preceding year. A positive value indicated increased percent cover between years while a negative value indicated a decrease in percent cover. The change in percent cover was then analysed in response to weather conditions of the preceding year. For example, the estimated percent cover in 2022 was subtracted from the estimated percent cover in 2021. The resulting change in percent cover value was then analysed in response to the average temperature and precipitation (see above) from 2021 (i.e. the preceding year).

### Statistical analysis

In order to make our data distributed more normally and have more constant variances, we transformed the percent cover data using the square root transformation ($\sqrt{x}$). All analyses were then conducted using this transformed data.

To analyse the change in percent cover in response to year, precipitation and temperature, we calculated Pearsons correlation coefficients in R (version 4.2.2) (R Development Core Team 2023). Pearson correlation coefficients were calculated for all five dominant species as well as the community with the function *cor.test* in R. Finally, to further explore any impact that measurement date might have had on percent cover, we calculated the number of calendar dates between measurements. We compared these dates to the change in percent cover value using a Pearson correlation analysis.

## Results

### Effects of year on percent cover

**Figure 3.**
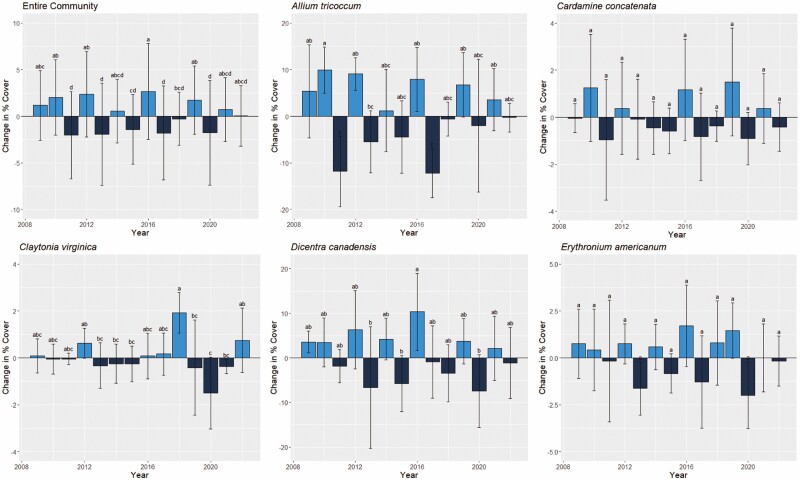
Change in percent cover over time for all five species and the total community. Different lowercase letters indicate significant differences in cover. Positive values indicate increases in cover; negative values indicate decreases in cover.

Year was not significantly correlated with change in percent cover (Table 1). We also did not find a correlation between change in percent cover and calendar days between surveys (*P* = 0.65, *r* = −0.02), indicating that sampling date did not impact observed percent cover (Supporting Information—Fig. S2). Finally, there was no obvious trend of increasing or decreasing cover through time, either for the community as a whole or for individual focal species (Fig. 3).

**Table 1.** Results of the Pearson correlation between the year and the change in percent cover. No years were significantly correlated with percent cover. Degrees of freedom for the community cover were (13, 754), all others (13, 82).

	Year	
Species	r	*P*
*Allium tricoccum*	−0.07	0.539
*Cardamine concatenata*	−0.07	0.522
*Claytonia virginica*	−0.18	0.614
*Dicentra canadensis*	−0.06	0.102
*Erythronium americanum*	−0.08	0.459
Community	−0.07	0.067

### Effects of temperature on percent cover

**Figure 4.**
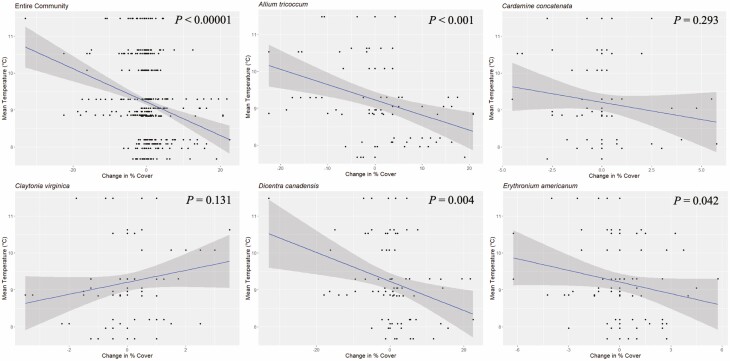
Year-over-year change in percent cover versus mean temperature (°C) for all five species and the total community. Here, mean temperature is the mean over the 62-day period in the spring prior to cover estimation. *P* values from Pearson correlation analyses are displayed on the graphs (see Table 2).

Temperature was significantly correlated with change in percent plant cover consistently across years (Table 2). Overall, cooler spring temperatures of one year were associated with increases in percent cover the following year, while warmer spring temperatures of one year were associated with decreases in percent cover the following year (Fig. 4). The exception was the change in percent cover for *C. virginica*, which had a positive correlation with the preceding year’s temperature, although this relationship was not significant. Temperature was also not significantly correlated with a change in cover for *C. concatenata.*

**Table 2.** Results of the Pearson correlation between previous-year mean temperature and change in percent cover as well as previous-year mean precipitation and change in percent cover. Bold *P* values are significant at *P* ≤ 0.05. Degrees of freedom for the community cover were (1, 754), all others (1, 82).

Species	Temperature		Precipitation	
	*r*	*P*	*r*	*P*
*Allium tricoccum*	−0.37	**<0.001**	0.21	0.058
*Cardamine concatenata*	−0.12	0.293	−0.01	0.899
*Claytonia virginica*	0.17	0.131	0.23	**0.035**
*Dicentra canadensis*	−0.31	**0.004**	0.07	0.521
*Erythronium americanum*	−0.22	**0.042**	0.12	0.261
Community	−0.20	**<0.00001**	0.11	**0.002**

**Figure 5.**
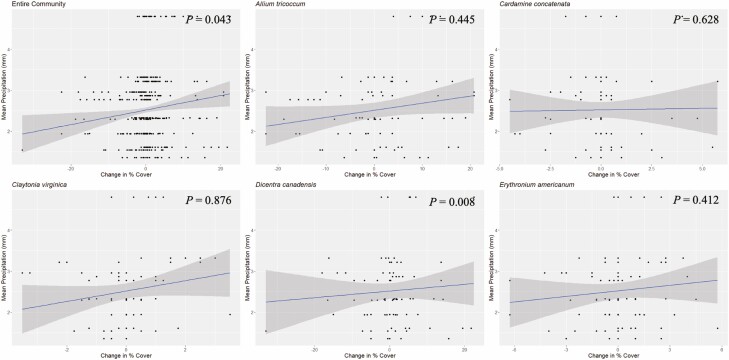
Percent cover versus same-year mean temperature (°C) for all five species and the total community. Here, mean temperature is the mean over the 30-day period in the same spring as the cover estimation. *P* values from Pearson correlation analyses are displayed on the graphs (see Table 3).

Temperature was significantly correlated with percent cover within the same year, but only for *D. canadensis* and the community (Table 3). These results trended in the opposite direction of the prior year’s weather results. Overall, warmer temperatures in the same year were correlated with greater cover (Fig. 5), but this relationship had fewer significant effects than the prior year’s analysis. Temperature from the same year was not significantly correlated with *A. tricoccum, C. concatenata, C. virginica* and *E. americanum.*

**Table 3.** Results of the Pearson correlation between same-year mean temperature and percent cover as well as same-year mean precipitation and percent cover. Bold *P* values are significant at *P* ≤ 0.05. Degrees of freedom for the community cover were (1, 808), all others (1, 88).

Species	Temperature		Precipitation	
	*r*	*P*	*r*	*P*
*Allium tricoccum*	0.08	0.445	−0.26	**0.014**
*Cardamine concatenata*	0.05	0.628	−0.06	0.600
*Claytonia virginica*	−0.02	0.876	0.15	0.153
*Dicentra canadensis*	0.28	**0.008**	−0.05	0.630
*Erythronium americanum*	0.09	0.412	0.03	0.793
Community	0.07	**0.043**	−0.04	0.205

### Effects of precipitation on percent cover

**Figure 6.**
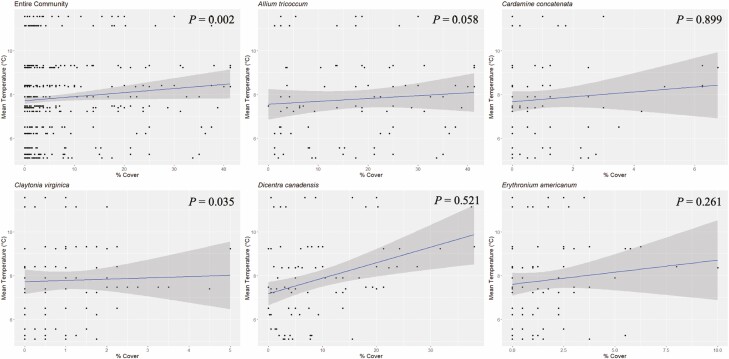
Year-over-year change in percent cover versus mean precipitation (mm) for all five species and the total community. Here, mean precipitation is the mean over the 62-day period in the spring prior to cover estimation. *P* values from Pearson correlation analyses are displayed on the graphs (see Table 2).

Precipitation the prior year was significantly correlated with a change in percent cover at the community level and with *C. virginica* (Table 2). It was marginally significant for *A. tricoccum*, (*P* = 0.058; Table 2). It was not significant for any remaining individual focal species. Despite this, all focal species and the community showed a positive relationship between change in percent cover and prior year precipitation, which indicates a trend of increasing percent cover with higher precipitation in the previous year (Fig. 6).

Precipitation was only significantly correlated with percent plant cover within the same year for *A. tricoccum* (Table 3). It did not significantly impact the overall community or the other individual species. Overall, these results trended in the opposite direction of the prior year’s weather results: increased precipitation in the same year was correlated with less cover (Supporting Information—Fig. S3). Some species, like *C. virginica*, showed an opposite trend, but this was not significant.

## Discussion

Spring ephemerals have the unique ability of growing in cooler temperatures before the other plant species that occupy their same area (Lapointe 2001; Dion *et al.* 2017). The literature is full of studies that have associated cooler temperatures with increases in spring ephemeral growth (Routhier and Lapointe 2002; Lapointe and Lerat 2006; Badri *et al.* 2007; Bernatchez and Lapointe 2012) and reproduction (Nault and Gagnon 1993; Kim *et al.* 2015; Sunmonu and Kudo 2015; Matthews and Mazer 2016). The results of the current study further support this idea, finding that cooler spring temperatures are associated with an increase in ephemeral plant cover at both the community and the individual species level. However, our study also found that cooler temperatures had the most significant and consistent impact on spring ephemeral cover the year after the ephemerals were growing in cooler conditions, not within the same year. Years following a spring with higher precipitation also saw a significant increase in cover for the entire community and for *C. virginica*. It is clear that the weather conditions spring ephemerals grow in will significantly impact their aboveground cover in the years that follow more than they would within the same year. Despite the current literature only consisting of studies that took place over a one-year timeframe, many of their findings provide support for the results of the current study. Nault and Gagnon observed ideal spring weather conditions for *A. tricoccum* reproduction in Canada: a warm April followed by a cool and wet May (1993). Nault and Gagnon’s study found that the warmer April temperatures allowed for early and rapid epigeous growth, while the wet, cool May delayed canopy closure. This extended high light period delayed *A. tricoccum* senescence, therefore extending their window for carbon gain (Nault and Gagnon 1993). As a result, Nault and Gagnon observed an increased seed set later in that same summer. Although our study did not examine reproduction, factors that increase plant flower and seed set are also likely to result in increases in growth and carbon storage below ground and would most likely lead to increased cover the following year. In fact, the below-ground storage and perennial organ most likely reflects the conditions of the previous growing season, and conditions that favour growth of the perennial organ would also result in greater plant coverage the following season. It is likely, then, that the reason why our study found previous-year weather conditions to be so important, as compared to other studies that found significant impacts from same-year weather conditions, is that we examined a different trait of spring ephemerals. It is known that both *A. tricoccum* and *E. americanum* leaf size are influenced by perennial organ size, which, in turn, is determined by previous-year weather conditions (Nault and Gagnon 1993; Lapointe 2001). Focusing on a trait that is documented as spanning several growing seasons uncovered the influence that previous-year conditions can have on these ephemerals. Regardless of trait, short-term studies like Nault and Gagnon’s (1993) all agree that cooler temperatures benefit spring ephemerals (Routhier and Lapointe 2002; Lundmark *et al.* 2009; Gandin *et al.* 2011; Kim *et al.* 2015; Sunmonu and Kudo 2015; Dion *et al.* 2017; Bertrand and Lapointe 2022). Where they disagree, however, is the reason colder temperatures are beneficial.

One potential reason for temperature effects on spring ephemerals is related to changes in canopy closure. Warmer temperatures are known to advance tree canopy closure, therefore reducing the photosynthetic period and hastening leaf senescence of spring ephemerals (Lee *et al.* 2022). Many forest wildflowers are considered light limited (Lapointe 2001; Whigham 2004) and early canopy closure can reduce an already short photosynthetic window. For example, *Podophyllum peltatum* (mayapple) has been found to be light, rather than nutrient or carbon, limited (Constable *et al.* 2007) and populations often do not display growth changes even when nutrient availability is increased (Burke *et al.* 2023). *Trillium erectum* leaf out has been shown to mismatch with sugar maple tree leaf out and canopy closure under warmer spring conditions, therefore reducing the carbon-gaining window of *T. erectum* and decreasing investments in reproduction as a result (Routhier and Lapointe 2002). *A. tricoccum* has been observed to grow better in areas with higher light availability due to delayed canopy closure (Dion *et al.* 2017). Under early canopy closure, *Erythronium japonicum* ephemerals allocate the majority of their resources to reproduction, resulting in a smaller number of low-quality seeds and potential population reductions in subsequent years (Kim *et al.* 2015). In sum, these studies suggest that cooler temperatures benefit spring ephemerals because they delay canopy closure, giving the ephemerals more time for resource acquisition, growth and reproduction.

On the other hand, it has also been suggested that temperature itself, rather than light availability, leads to early senescence of spring ephemerals (Lapointe 2001). Warmer temperatures alone can lead to reductions in growth of belowground organs (e.g. bulbs) and induce a reduction in the belowground carbon sink leading to earlier leaf senesce (Lapointe 2001). Lundmark *et al.* found that warm temperatures negatively affect both carbon metabolism and cell growth in the perennial organ of *Crocus vernus* (2009). *Gagea lutea* plants do not experience an advantage with prolonged high light conditions, as seed set rates do not differ from that of shaded *G. lutea* plants (Sunmonu and Kudo 2015). Bulb biomass, leaf longevity and pollen production of *G. lutea* are also all reduced when grown in warmer spring temperatures (Sunmonu and Kudo 2015). *E. americanum* does not appear to benefit from delayed leaf senescence, as the plants are unable to accumulate more carbon in their perennial bulb during this extended period (Bertrand and Lapointe 2022). When grown at low temperatures, however, *E. americanum* plants produce larger perennial bulbs because they are less source-sink imbalanced (Gandin *et al.* 2011). Clearly, many ephemeral species’ ability to display epigeous tissues and acquire carbon is, at least in part, dependent upon temperature itself.

Currently, multi-year studies that investigate temperature and precipitation focus primarily on phenology and mismatch with the tree canopy (Heberling *et al.* 2019; Lee *et al.* 2022). Our study did not assess canopy openness, nor did it assess carbon acquisition, so one explanation cannot be favoured over the other. These two ideas are closely linked, and it is possible that it could be a combination of canopy openness and increased storage at cooler temperatures that affect spring ephemeral growth and changes in cover from one year to the next. As the effects of climate change develop further, understanding the mechanisms behind the response of spring ephemeral species to warmer temperatures is critical to predicting how these species will fare.

In contrast to temperature, precipitation had less of an influence on future cover of spring ephemerals, but we did see some significant effects. The previous years’ precipitation did affect coverage of spring ephemerals the following year for the overall community and for *C. virginica*, which only responded to precipitation and not temperature. This is not surprising, as *C. virginica* has been recorded as being able to grow without a cold period to break dormancy (Risser and Cottam 1967), although it is not completely insensitive to temperature (Xie *et al.* 2022). *A. tricoccum* was the only species that did respond to precipitation within the same year of measurement. Although most of our measured species seemed insensitive to precipitation when analysed separately, there is reason to believe that temperature and precipitation may interact to affect communities. For example, Matthews and Mazer (2016) found an interaction between temperature and precipitation on the flowering phenology of *T. ovatum* based on analysis of herbarium specimens. In this study, increased precipitation appeared to significantly delay flowering of *T. ovatum*, but this delay was the most significant when the minimum spring temperatures were the coolest (Matthews and Mazer 2016). To our knowledge, very few studies in the literature have analysed the interaction between temperature and precipitation (Fu *et al.* 2014; Matthews and Mazer 2016) but this should be an area of future research.

Future studies should also consider precisely tracking spring leaf development to determine true maximum expansion. We visually monitored leaf expansion and surveyed percent cover when peak expansion was visually confirmed. Our percent cover data were not significantly impacted by survey date, which speaks to the strength of our observations as currently reported. However, monitoring degree days and individual leaf expansion would allow for surveys to be conducted at the most accurate time possible and potentially uncovering additional information on how species are influenced by previous weather conditions. Finally, it is important to note that the increase in cover observed in the present study does not necessarily represent an increase in growth. Many species of spring ephemerals, including several studied here, exhibit clonal growth. *A. tricoccum* bulbs divide during reproductive years, giving the impression of greater plant coverage due to the increase in plant number (Nault and Gagnon 1993). *E. americanum* reproduce via a combination of sexual reproduction and clonality, but typically this clonal reproduction is lower than in other ephemeral species (Stokes *et al.* 2019). *D. canadensis* plants use clonal reproduction to maximize fitness when flowering individuals are disadvantaged by habitat degradation and other energy costs (Lin *et al.* 2016). The changes in cover we observed, then, could be the result of increased bulb division, leaf growth or greater reproduction. The confines of the present study preclude us from making this distinction, but all could easily explain the coverage differences seen here. Future research should aim to address this gap in knowledge.

## Conclusion

Current multi-year studies on spring ephemerals of deciduous forests largely explore the phenological mismatch of growth and reproduction with tree canopy closure (Heberling *et al.* 2019; Lee *et al.* 2022). Photoperiod, as related to canopy closure, is important for spring ephemeral growth as it affects the window for photosynthesis and carbon gain (Routhier and Lapointe 2002) and the effect of temperature influences the length of this high light period since it affects the timing of canopy closure (Richardson *et al.* 2006). However, there is also evidence to suggest that temperature alone has a direct effect on carbon storage for spring ephemerals, where higher temperatures result in lower belowground carbon storage (Gandin *et al.* 2011). The data reported in the current study support this idea, where warmer spring temperatures of the prior year consistently led to reductions in cover of spring ephemerals in the following year on a dataset that spanned more than a decade. This suggests that belowground carbon storage was reduced in years with warmer spring temperatures, which led to lower overall plant cover in the following year. It is important to note that the current study did not assess canopy openness. Thus, the higher spring temperatures of the prior year could have led to reductions in spring ephemeral cover as a result of early canopy closure (and thus reduced light availability); however, this cannot be assessed with the current data set. Nonetheless, the data reported here offer an interesting finding of prior year temperature influencing spring ephemeral growth. We suggest in future research that previous weather conditions and canopy closure be explored as potential environmental drivers of growth and reproduction of spring ephemerals.

## Supporting Information

The following additional information is available in the online version of this article –

Figure S1. View from long-term plot 1A. Photo taken 21 April 2021.

Figure S2. Calendar days between surveys versus change in percent cover for the entire community. Surveying earlier or later from one year to the next did not impact the change in cover (*r* = −0.02, *P* value = 0.65).

Figure S3. Percent cover versus same-year mean precipitation (mm) for all five species and the total community. Here, mean precipitation is the mean over the 30-day period in the same spring as the cover estimation. *P* values from Pearson correlation analyses are displayed on the graphs (see Table 3).

plad078_suppl_Supplementary_Figures_1-3

plad078_suppl_Supplementary_Data

## Data Availability

The data and R code underlying this article are available in the online Supporting Information.
