# Supplementary material for: Interannual variation in spring weather conditions as a driver of spring wildflower coverage: a 15-year perspective from an old-growth temperate forest
Source: AoB Plants. 2023 Nov 14;15(6):plad078. doi: 10.1093/aobpla/plad078 (PMC10727473; doi:10.1093/aobpla/plad078)
Supplement: plad078_suppl_Supplementary_Figures_1-3 [file plad078_suppl_supplementary_figures_1-3.pdf]

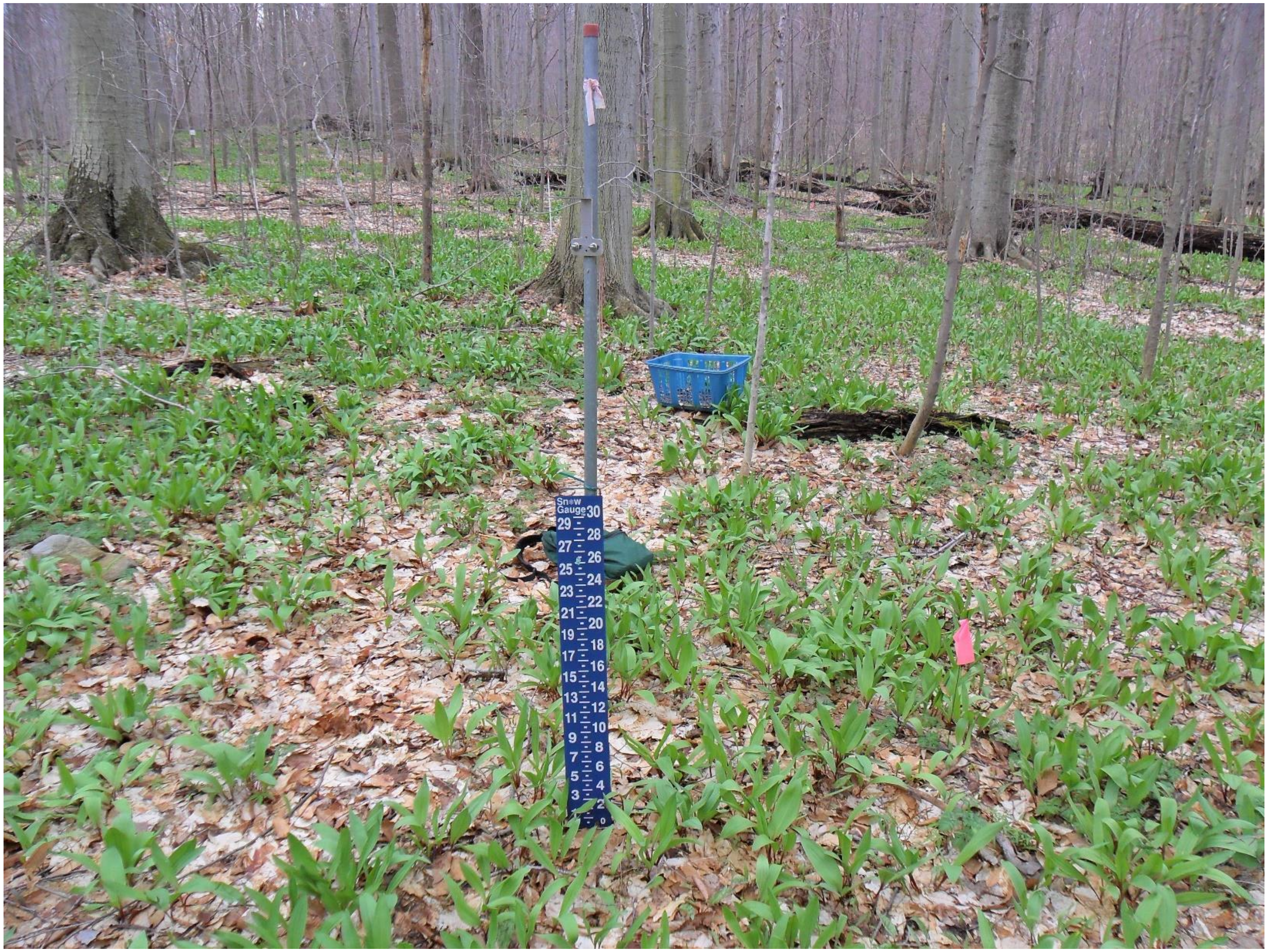

Supporting Information Figure 1. View from long-term plot 1A. Photo taken April 21, 2021

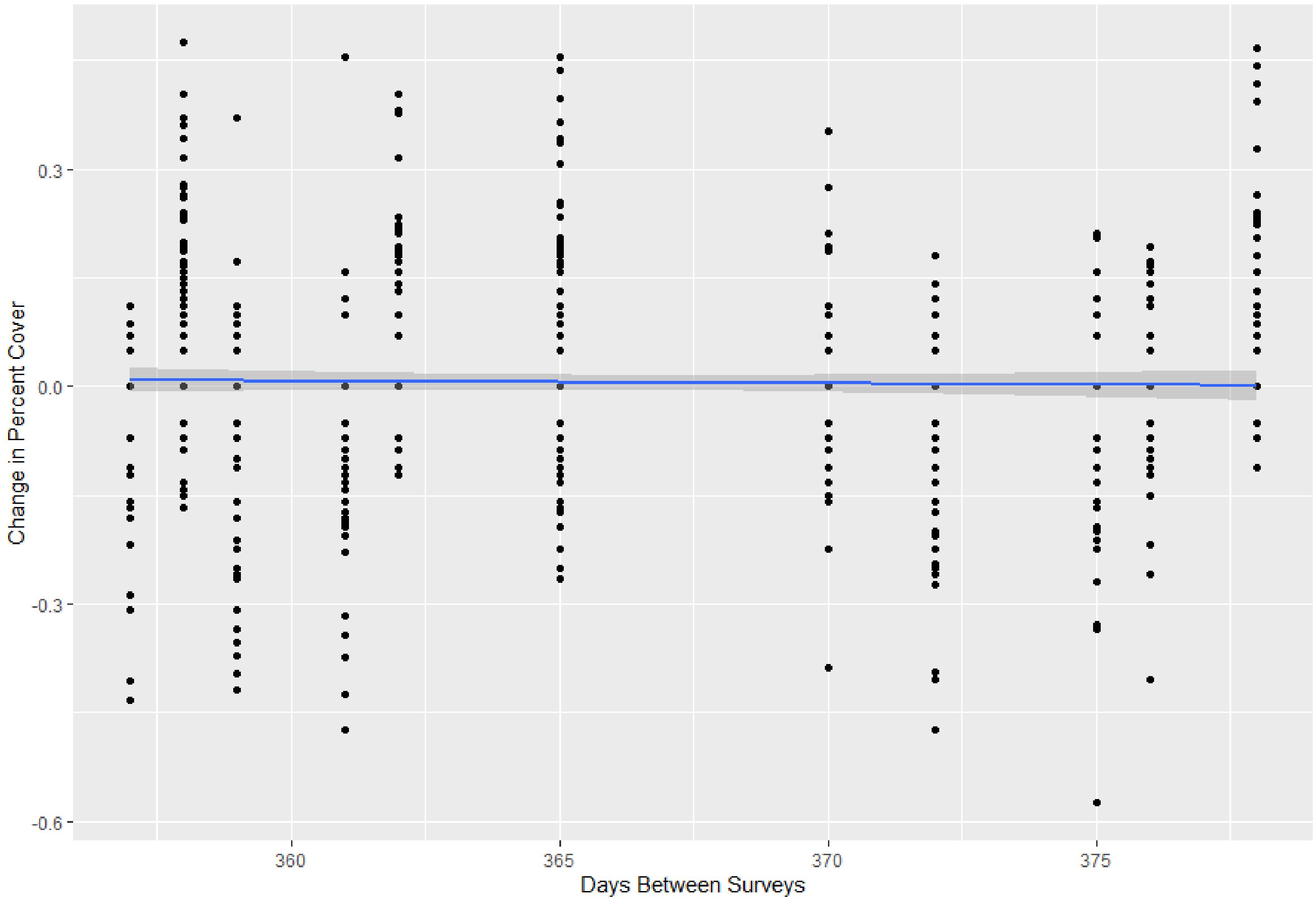

Supporting Information Figure 2. Calendar days between surveys vs. change in percent cover for the entire community. Surveying earlier or later from one year to the next did not impact the change in cover (r = -0.02, *P*-value = 0.65).

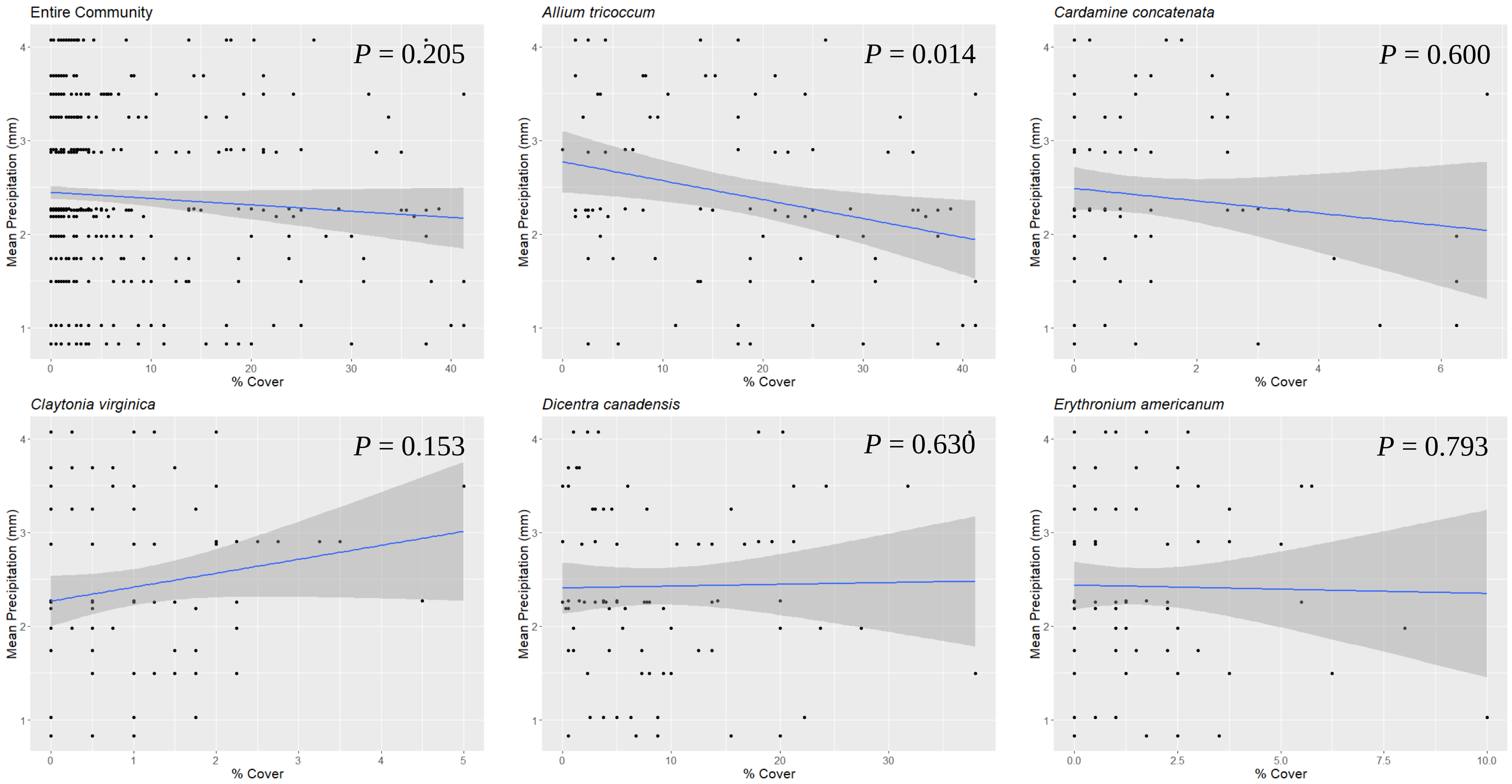

Supporting Information Figure 3. Percent cover vs. same year mean precipitation (mm) for all five species and the total community. Here, mean precipitation is the mean over the 30-day period in the same spring as the cover estimation. P-values from Pearson correlation analyses are displayed on the graphs (see Table 3).
